# Best practices for expansion of smoke-free and aerosol-free environments in Europe: Protocol for the consultation to experts

**DOI:** 10.18332/tpc/192786

**Published:** 2024-10-18

**Authors:** Dolors Carnicer-Pont, Anna Mar López Luque, Biljana Kilibarda, Milena Vasic, Melinda Penzes, Chiara Stival, Adrian Gonzalez, Helena Koprivnikar, Giulia Carreras, Giuseppe Gorini, Irene Possenti, Alessandra Lugo, Silvano Gallus, Esteve Fernández

**Affiliations:** 1 Tobacco Control Unit, Catalan Institute of Oncology – WHO Collaborating Centre for Tobacco Control, l’Hospitalet de Llobregat, Catalonia, Spain; 2 Tobacco Control Research Group, Bellvitge Biomedical Research Institute, l’Hospitalet de Llobregat, Catalonia, Spain; 3 Centre for Biomedical Research in Respiratory Diseases, Institute of Health Carlos III, Madrid, Spain; 4 Institute of Public Health of Serbia “Dr Milan Jovanovic Baut”, Belgrade, Serbia; 5 Health Services Management Training Centre, Semmelweis, Hungary; 6 National Koranyi Institute of Pulmonology, Budapest, Hungary; 7 Department of Medical Epidemiology, Istituto Di Ricerche Farmacologiche Mario Negri IRCCS, Milan, Italy; 8 Group of Evaluation of Health Determinants and Health Policies, Faculty of Medicine and Health Sciences, Basic Sciences Department, Universitat Internacional de Catalunya, Catalonia, Spain; 9 National Institute of Public Health, Ljubljana, Slovenia; 10 Institute for Cancer Research, Prevention and Clinical Network (ISPRO), Florence, Italy; 11 Department of Clinical Sciences, Faculty of Medicine and Health Sciences, University of Barcelona, Barcelona, Spain

**Keywords:** protocol, smoke-free, aerosol-free environments, secondhand smoke, secondhand aerosol, best practice, clean air policies

## Abstract

Smoke-free legislation has been shown to positively impact reducing secondhand smoke (SHS) exposure, especially in countries that have implemented comprehensive legislation rather than partial bans. Also, secondhand aerosols (SHA) that come from the heating of tobacco or liquids, with or without nicotine, in electronic nicotine delivery systems (ENDS) have been proven to increase levels of harmful substances in the air. Therefore, protection against SHS and SHA exposure and expansion of smoke- and aerosol-free environments (SAFE) should be taken into account when creating or trying to expand or enforce clean air policies. This article aims to present the protocol for a consultation with experts on tobacco and nicotine control in order to identify best practices, barriers, and opportunities for the expansion of SAFE in Europe. We identified experts among policymakers, researchers, and tobacco regulators in European countries and invited them to participate in the consultation by completing an online survey designed, programmed, and pilot-tested using Survey Monkey. The responses to the questionnaire contained quantitative and qualitative information that was thematically analyzed. The experts’ consultation allowed us to produce a report on barriers and opportunities for SAFE, a report and a position paper on SAFE best practices, a web-based repository of best practices, and a weight of evidence paper that assembles evidence supporting the expansion of SAFE on indoor and outdoor spaces.

## INTRODUCTION

Smoke-free legislation has been shown to be effective and have a positive impact on the population’s health^1^, as people who live in countries that have smoke-free bans are less exposed to secondhand smoke (SHS), especially if they have comprehensive legislation rather than partial bans^2^. Moreover, smoke-free legislation could also change behaviors beyond the ban itself, such as not smoking at home^3,4^ and reducing smoking prevalence, mostly among women^5^. However, not only traditional tobacco products and SHS should be considered when talking about smoke-free environments^6^. The use of electronic cigarettes (e-cigarettes) or heated tobacco products (HTPs) produces aerosols containing different hazardous substances^7,8^ that are exhaled by users as secondhand aerosols (SHA).

There is growing evidence supporting the health harm of SHA, which contains numerous toxic and carcinogenic substances^9,10^, Moreover, the use of e-cigarettes and HTPs increases levels of harmful substances in the air of enclosed places-^11,12,13,14^. However, most of the legislations in the WHO European Region are not comprehensive enough when it comes to e-cigarettes and HTPs^10^. Therefore, protection against SHA should be taken into account when creating or trying to expand or enforce clean air policies^8^.

Another challenge is the lack of legislative solutions regarding smoke- and aerosol-free environments (SAFE). Regardless of some common regional regulation, such as the Tobacco Products Directive (TPD)^15^ in the European Union (EU) or, more globally, the WHO Framework Convention for Tobacco Control (FCTC)^16^, the level of protection offered to non-smokers varies depending on the country they live, and this is mainly a consequence of differences between clean air policies across countries^2^. Additionally, we must take into account that there are also differences in the terms of compliance and enforcement of these legislations^17^.

As acknowledged in recent global reports on the tobacco epidemic and tobacco control^18,19^, nations are progressively expanding smoke-free regulations to encompass outdoor spaces. Despite the decline in SHS exposure attributable to the positive impact of effective legislation, substantial exposure still persists in certain public and private settings, such as bars and restaurants, or homes and cars^20^.

To support further progress in protection from SHA and SHS, Work Package 8 of the Second Joint Action for Tobacco Control (JATC2) aimed to outline and disseminate best practices in order to address the upcoming challenges for smoke-free environments in Europe. For this purpose, a consultation with European experts on best practices, barriers, and opportunities to expand SAFE was designed.

This article aims to present the protocol used to identify best practices, barriers, and opportunities to protect people from exposure to SHS and SHA produced by e-cigarettes, HTPs, and other tobacco or nicotine products.

## METHODOLOGICAL APPROACH

### Identification and selection of experts

We applied several methods to identify and involve tobacco control experts across Europe in our consultation. First, the JATC2 employed a contact list of all partners and their affiliated authorities, institutes, or organizations working in the field of tobacco control (policymakers, regulators, researchers, tobacco inspectors, NGO activists) partners from EU Member States, as well as non-EU countries of Europe. Second, the Catalan Institute of Oncology, a WHO Collaborating Centre for Tobacco Control, provided its list of contacts, including speakers and attendees, to five editions of five ICO-WHO Symposia on tobacco control. Third, the Smoke-Free Partnership (SFP) and the European Network for Smoking and Tobacco Prevention (ENSP) were requested to provide their list of contacts, partners, and members for the JATC2 consultation. From all of these sources, we identified 110 experts from 30 European countries (27 EU Member States, Norway, Serbia, and the United Kingdom).

### Inviting experts to participate in the consultation

All of the identified experts were invited by e-mail to participate in the consultation. The invitation email explained the objectives of the consultation, the instructions to complete the online questionnaire, and the links to access both Section 1 and Section 2. After accepting the invitation for the consultation, the experts were sent the online questionnaire gathering information on any type of SAFE, including both public and private environments and outdoor and enclosed places. After acceptance, the experts were sent an online questionnaire gathering information on any type of smoke-free environment, including both private and public environments, outdoor and enclosed places, and protection from tobacco smoke or exposure to aerosols from HTPs or e-cigarettes. Of the 110 invited experts, 61 (response rate of 55.4%) from 29 EU countries (Austria, Belgium, Croatia, Cyprus, Czech Republic, Denmark, Estonia, Finland, France, Germany, Greece, Hungary, Ireland, Italy, Latvia, Lithuania, Luxemburg, Malta, Netherlands, Norway, Poland, Portugal, Romania, Serbia, Slovakia, Slovenia, Spain, Sweden and United Kingdom of Great Britain and Northern Ireland) provided full or partial answers to the online questionnaire (Supplementary file)

### Designing, programming, and testing the online questionnaire

The online questionnaire^21^ contained a compulsory information and consent form, as well as two other sections. Section 1, with 26 questions (nine on sociodemographic information, 9 open-ended, and 8 open questions), explored the comprehensiveness of existing smoke- and aerosol-free legislation, perceived compliance, perceived barriers, and opportunities for expansion. The first section also explored the extent of tobacco industry interference. Section 2 included 42 questions: 16 open-ended and 26 open questions, allowing national experts to provide detailed information through text or links about practices that are considered best practices on SAFE. The online questionnaire was programmed using Survey Monkey. It included multiple choice quantitative closed-questions and qualitative open-ended questions and also allowed the attachment of documents or links to some questions. Testing of the questionnaire was conducted by the WP8 partners, who, after filling it in, provided feedback about the duration, readability, and comprehensibility of the questionnaire.

### Completing the survey filling in and data collection

Each respondent was asked to report information on up to four best practices and to provide four specific online links for each best practice. Since the questionnaire had more than 60 questions and many of them required detailed descriptions, the process of filling it in required a median of two days (two entries) for most of the respondents. The questionnaire was structured to enable experts to pause, save, and resume completion at the precise point where it was initially suspended.

The follow-up of data collection was conducted weekly by programmed routines in Survey Monkey, including follow-up of the status of completion of questionnaires for each expert and reminders to continue with the task. The questionnaire was accessible for a duration of up to 12 weeks from its launch date on the 15 June up to the 15 August 2022.

### Data handling and record keeping

All data were stored in Excel and transferred to Stata for analysis. A web-based repository with all best practices on SAFE reported was created and is currently fully functional.

### Data analysis

*Step 1*

We assessed response rate, namely the number of answers received (total and per country; % of non-response); the number of Best Practices (BP) received per country; completeness of received questionnaires (overall %; and question-specific details) and the correctness of links and/or documents provided.

*Step 2*

Descriptive analyses were conducted to explore the distribution of BP topics. The quantitative variables of the practices, barriers, and opportunities and each category are presented in Tables 1 and 2.

**Table 1:** Quantitative variables to assess best practices on SAFE

*Variables*	*Categories*
**Type of practice**	Information/awareness raising program Policy Action plan Regulation/ban Monitoring/surveillance Service delivery approach/method Tool/instrument Guideline Training E-health – mHealth Health in all policies Don’t know
**Phase of the practice**	Practice is at the first stage of implementation but not yet totally developed Practice has been developed/adopted but not yet enforced Practice has been implemented (enforced/promoted) Practice has been evaluated Practice has been registered in a best practice registering portal Don’t know
**Responsibility for the practice**	Municipality/city Province/region Nation Public agency University Government NGOs Private institution Don’t know
**Duration of the practice**	Practice is ongoing Practice has ended Don’t know
**Start and end date of the practice**	
**Scope of the practice**	International National Regional Local
**Funding of the practice**	Own resources External resources – public External resources – private excluding the tobacco or nicotine industry External resources – private including the tobacco or nicotine industry No funds required Don’t know
**Objectives of the practice**	Smoke-free indoor settings (conventional tobacco products) Smoke-free outdoor settings (conventional tobacco products) Voluntary home smoking ban (conventional tobacco products) Car smoking ban with minors or pregnant women (conventional tobacco products) Car smoking ban also without minors or pregnant women (conventional tobacco products) Smoking ban as an anti-COVID-19 measure Indoor aerosol-free regulation for e-cigarettes Outdoor aerosol-free regulation for e-cigarettes Voluntary home aerosol ban regulation for e-cigarettes Car vaping ban with minors or pregnant women Car vaping ban also without minors or pregnant women Vaping ban as an anti-COVID-19 measure Indoor aerosol-free regulation for heated tobacco products Outdoor aerosol-free regulation for heated tobacco products Voluntary vaping ban regulation for heated tobacco products Car heated tobacco product ban with minors or pregnant women Car heated tobacco product ban also without minors or pregnant women Ban of heated tobacco products use as an anti-COVID-19 measure
**Target settings**	Restaurants and bars (indoor) Hotels (indoor) Train stations and public transports (indoor) Airports (indoor) Workplace (indoor) Schools/public education institutions/educational venues except universities (indoor) Universities (indoor) Cinemas/theatres (indoor) Hospitals including outpatient clinics (indoor) Primary health care institutions (indoor) Institutions from social sector (indoor) Prisons (indoor) Cars Home Restaurants’ patios/terraces (outdoor) Bus, tramway, trolley-bus stop waiting areas (outdoor) Parks (outdoor) Underpass (outdoor) Stadiums and outdoor arenas (outdoor) Beaches (outdoor) Outdoor areas of hospitals and healthcare institutions (outdoor); Outdoor areas of school (outdoor) Children’s playgrounds (outdoor)
**Target population**	General population Gender specific groups Age specific groups Socioeconomic position (including educational level) Certain levels in education system Cultural/ethnic background Vulnerable groups (Disability) Vulnerable groups (Diseases) Vulnerable groups (Prisoners) Vulnerable groups (Sexual diversity; e.g. LGBTQ) Vulnerable groups (Pregnant women) Vulnerable groups (Immigrants/refugees) Urban setting Rural settings Don’t know
**Involvement of the target population in development; implementation or evaluation of the practice**	International/European public health authorities National public health authorities Regional public health authorities Local public health authorities Hospital staff Primary care center staff Specialized physicians General practitioners Pharmacists Nurses Other health care professionals Informal caregivers Researchers/academics School/kindergarten teachers Employers/employees Civil society
**Enforcement of the practice**	Yes No
**Evaluation of the practice**	Yes, by an external partner Yes, the evaluation was carried out internally Not yet, the intervention is still ongoing but the evaluation is foreseen No Don’t know
**Transferability of the practice**	Transferability has not been considered Practice has been implemented on local/regional/national level and transferability has not been considered in a systematic way Ready for transfer, but the practice has not been transferred yet Practice has been developed on local/regional/national level and transferability has been considered and structural, political and systematic recommendations have been presented. However, the practice has not been transferred yet Practice has been transferred (i.e. scaled-up) within the same country/region Practice has been scaled-up to other locations or regions or at national scale in the same country
**Sustainability**	Practice has institutional support and stable human resources Practice provides training of staff in order to sustain it A sustainability strategy has been developed None of the above options

**Table 2:** Quantitative variables to assess barriers and opportunities for expansion of compliance with and enforcement of SAFE

*Variables*	*Response categories*
In your country, can you identify any **barriers** for the **expansion** of smoke and aerosol-free environment policies?	Yes/no
In your country, can you identify any barriers to the improvement of **compliance** with (or enforcement of) smoke and aerosol-free environment policies?	Yes/no
In your country, to what extent do you think the tobacco or nicotine industries (and their allies) are interfering with the **expansion** of smoke and aerosol-free environments?	No interference Small Moderate Large Very large interference
In your country, to what extent do you think the tobacco or nicotine industries (and their allies) are interfering with the **enforcement** of smoke and aerosol-free environments?	No interference Small Moderate Large Very large interference
In your country, can you identify any **opportunities** for the **expansion** of smoke and aerosol-free environment policies?	Yes/no
In your country, can you identify any **opportunities** for the improvement of **compliance** with (or enforcement of) smoke and aerosol-free environment policies?	Yes/no

### Ethical considerations

Informed consent was obtained by ticking affirmatively the two final questions of the first page of the online questionnaire: 1) ‘I understand and agree that the provided information is correct and may be used by the WP8 leaders for the purposes indicated’; and 2) ‘I understand and agree that my name and institution can be listed in the JATC-2 website and reports. Experts had the right to withdraw at any point of the consultation.

### Conflicts of interest

All the experts were asked about potential conflicts of interest with the aim of the consultation. No experts with links to the tobacco and/or electronic cigarette industry were included in the experts’ panel.

## DISCUSSION

Quantitative variables addressing best practices and allowing multiple choice responses were analyzed as a number of responses (frequencies) for each category of the variable. A number of practices and percentages were calculated for the single-choice response variables. Qualitative open-ended questions addressed best practices, and careful reading of each practice allowed the classification of the practice into groups. A series of thematic analyses were conducted using subjective coding systems by three members of Work Package 8 of the Joint Action on Tobacco Control 2. Responses were categorized thematically using the Title of the practice as a reference to group the practices into a broader group ‘type of setting’. Finally, the list and details of each practice were placed in a Web-based repository.

Qualitative open-ended questions addressing barriers and opportunities, a series of thematic analyses were conducted using subjective coding systems by three members of Work Package 8 of the Joint Action on Tobacco Control 2. Responses were first categorized thematically; however, this classification resulted in a high number of categories (n=11–15) that were difficult to overview. Therefore, as a second step, we collapsed these into broader thematic categories (n=5–6). Finally, these categories were presented in tables as numbers and percentages of the total responses, taking the total number of experts as the denominator.

Additional information on best practices was obtained by web links and PDF documents uploaded while answering the survey. We conducted best practices content analysis and summarized it in a narrative report to allow synthesis and readability of the results.

## CONCLUSIONS

This protocol has been a guide for all the foreseen activities related to the identification of best practices to expand SAFE in European countries. It allowed us to systematically work on the online consultation with experts^22^, the report of the symposium with experts, the reports on best practices^23^, barriers, and opportunities for SAFE^24^, the web-based repository^25^, the weight of evidence^26^, and finally, the position paper for SAFE^27^.

## Supplementary Material



## Data Availability

The data supporting this research are available from the authors on reasonable request.
