# Supplementary material for: Best practices for expansion of smoke-free and aerosol-free environments in Europe: Protocol for the consultation to experts
Source: Tob Prev Cessat. 2024 Oct 18;10:10.18332/tpc/192786. doi: 10.18332/tpc/192786 (PMC11487914; doi:10.18332/tpc/192786)
Supplement: Supplementary file 1 [file TPC-10-44-s1.pdf]

Supplementary table 1: Number of participating experts by position and country

	Governmental	NGO	Public hospital	Public research centre	University	Total
**Austria**	2	1				3
**Belgium**	1	2				3
**Croatia**	1				1	2
**Cyprus**	3					3
**Czech Republic**	3					3
**Denmark**		1				1
**Estonia**	3					3
**Finland**	1					1
**France**	1	1				2
**Germany**		1		1		2
**Greece**	1			1		2
**Hungary**	3					3
**Ireland**	2	1				3
**Italy**	1	1				2
**Latvia**	3					3
**Lithuania**		1				1
**Luxemburg**	1	1				2
**Malta**	1					1
**Netherlands**	2	1		1		4
**Norway**	1	1				2
**Poland**					1	1
**Portugal**					2	2
**Romania**			1			1
**Serbia**	1					1
**Slovakia**	2					2
**Slovenia**	2					2
**Spain**		1				1
**Sweden**	2		1			3
**United Kingdom**					2	2
**Total**	37	13	2	3	6	61

©2024 Carnicer-Pont D. et al.
